# Supplementary material for: Causal Relationship between Aging and Anorexia Nervosa: A White-Matter-Microstructure-Mediated Mendelian Randomization Analysis
Source: Biomedicines. 2024 Aug 16;12(8):1874. doi: 10.3390/biomedicines12081874 (PMC11351342; doi:10.3390/biomedicines12081874)
Supplement: Supplementary file 1 [file biomedicines-12-01874-s001.zip › Supplementary Figure/Figure S4/Singe SNP plot of MR analysis for TL to ubm-b-1459.pdf]

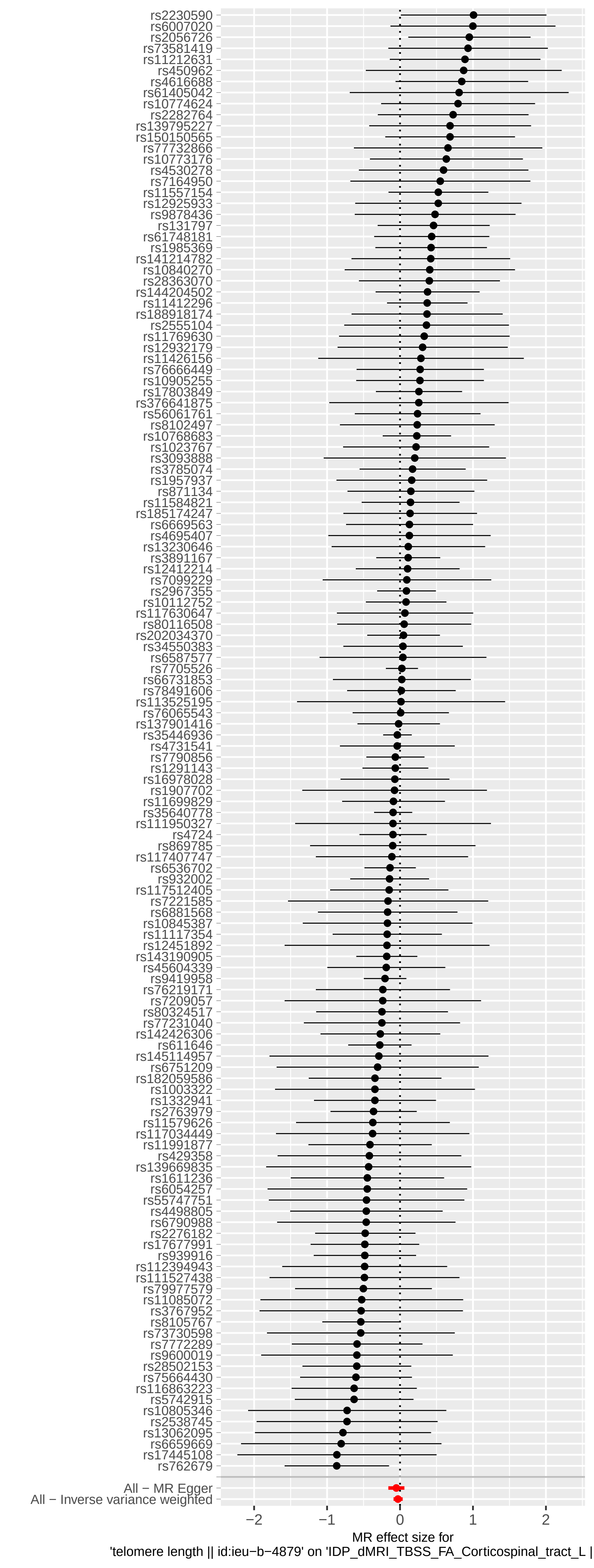
rs2230590
rs6007020
rs2056726
rs73581419
rs11212631
rs450962
rs4616688
rs61405042
rs10774624
rs2282764
rs139795227
rs150150565
rs77732866
rs10773176
rs4530278
rs7164950
rs11557154
rs12925933
rs9878436
rs131797
rs61748181
rs1985369
rs141214782
rs10840270
rs28363070
rs144204502
rs11412296
rs188918174
rs2555104
rs11769630
rs12932179
rs11426156
rs76666449
rs10905255
rs17803849
rs376641875
rs56061761
rs8102497
rs10768683
rs1023767
rs3093888
rs3785074
rs1957937
rs871134
rs11584821
rs185174247
rs6669563
rs4695407
rs13230646
rs3891167
rs12412214
rs7099229
rs2967355
rs10112752
rs117630647
rs80116508
rs202034370
rs34550383
rs6587577
rs7705526
rs66731853
rs78491606
rs113525195
rs76065543
rs137901416
rs35446936
rs4731541
rs7790856
rs1291143
rs16978028
rs1907702
rs11699829
rs35640778
rs111950327
rs4724
rs869785
rs117407747
rs6536702
rs932002
rs117512405
rs7221585
rs6881568
rs10845387
rs11117354
rs12451892
rs143190905
rs45604339
rs9419958
rs76219171
rs7209057
rs80324517
rs77231040
rs142426306
rs611646
rs145114957
rs6751209
rs182059586
rs1003322
rs1332941
rs2763979
rs11579626
rs117034449
rs11991877
rs429358
rs139669835
rs1611236
rs6054257
rs55747751
rs4498805
rs6790988
rs2276182
rs17677991
rs939916
rs112394943
rs111527438
rs79977579
rs11085072
rs3767952
rs8105767
rs73730598
rs7772289
rs9600019
rs28502153
rs75664430
rs116863223
rs5742915
rs10805346
rs2538745
rs13062095
rs6659669
rs17445108
rs762679
All − MR Egger
All − Inverse variance weighted
−2
−1
0
1
2
MR effect size for
'telomere length || id:ieu−b−4879' on 'IDP_dMRI_TBSS_FA_Corticospinal_tract_L |
